# Supplementary material for: Identification of an Immune-Related Prognostic Signature for Glioblastoma by Comprehensive Bioinformatics and Experimental Analyses
Source: Cells. 2022 Sep 26;11(19):3000. doi: 10.3390/cells11193000 (PMC9562700; doi:10.3390/cells11193000)
Supplement: Supplementary file 1 [file cells-11-03000-s001.zip › cells-1872351-supplementary.pdf]

Supplementary Table S1

	Forward (5’-3’ sequence)	Reverse (5’-3’ sequence)
GAPDH	GGAGCGAGATCCCTCCAAAAT	GGCTGTTGTCATACTTCTCATGG
C5AR1	ATCTTTGCAGTCGTCTTCCTG	CGGCTACCGCCAAGTTGAG
IL10	ATGCCCCAAGCTGAGAACCAAGA	GCTGGGTCAGCTATCCCAGAG
PPP4C	CTGGATCAGATTCGGACAATCG	CACCGTCTCATTGAAGTGCCA
